# Efficacy, feasibility and tolerability of ketogenic diet for the treatment of poor response to bariatric surgery

**DOI:** 10.1007/s40618-023-02034-2

**Published:** 2023-02-21

**Authors:** F. Vinciguerra, S. Longhitano, N. Carrubba, L. Piazza, C. Di Stefano, M. L. Arpi, R. Baratta, M. Hagnäs, L. Frittitta

**Affiliations:** 1 Department of Clinical and Experimental Medicine, University of Catania, Catania, Italy; 2 General and Emergency Surgery Department, Garibaldi Hospital, Catania, Italy; 3 Endocrinology Unit: Garibaldi Hospital, Catania, Italy; 4 Rovaniemi Health Center, Rovaniemi and Primary Health Care Unit, Lapland Central Hospital, Rovaniemi, Finland; 5 Medical Research Center Oulu, University of Oulu and Oulu University Hospital, Oulu, Finland; 6 Diabetes, Obesity and Dietetic Center, Garibaldi Hospital, Catania, Italy

**Keywords:** Very-low-calorie ketogenic diet, Bariatric surgery, Poor response, Insufficient weight loss, Weight regain

## Abstract

**Purpose:**

Poor response to bariatric surgery, namely insufficient weight loss (IWL) or weight regain (WR), is a critical issue in the treatment of obesity. The purpose of our study was to assess the efficacy, feasibility, and tolerability of very low-calorie ketogenic diet (VLCKD) for the management of this condition.

**Methods:**

A real-life prospective study was conducted on twenty-two patients who experienced poor response after bariatric surgery and followed a structured VLCKD. Anthropometric parameters, body composition, muscular strength, biochemical analyses, and nutritional behavior questionnaires were evaluated.

**Results:**

A significant weight loss (mean 14.1 ± 4.8%), mostly due to fat mass, was observed during VLCKD with the preservation of muscular strength. The weight loss obtained allowed patients with IWL to reach a body weight significantly lower than that obtained at the post-bariatric surgery nadir and to report the body weight of patients with WR at the nadir observed after surgery. The significantly beneficial changes in nutritional behaviors and metabolic profiles were observed without variations in kidney and liver function, vitamins, and iron status. The nutritional regimen was well tolerated, and no significant side effects were detected.

**Conclusion:**

Our data demonstrate the efficacy, feasibility, and tolerability of VLCKD in patients with poor response after bariatric surgery.

**Supplementary Information:**

The online version contains supplementary material available at 10.1007/s40618-023-02034-2.

## Introduction

Bariatric surgery represents one of the most effective strategies for the management of obesity, inducing significant weight loss with consequent amelioration of metabolic and cardiovascular comorbidities, such as amelioration of life expectancy [1, 2]. However, a substantial number of patients experience a poor response to bariatric surgery such as insufficient weight loss (IWL) or progressive weight regain (WR) after a successful primary procedure [3]. Both conditions can be considered long-term complications of bariatric surgery compromising its positive effects and favoring the persistence or recurrence of comorbidities (i.e., diabetes mellitus and hypertension) with negative consequences on the patient’s psychological and physical health [4, 5]. A poor response following bariatric surgery is multifactorial, including surgical, hormonal, metabolic, mental, and lifestyle conditions that can contribute to its occurrence [6, 7]. The management of these conditions is still unclear. The revision of the surgical procedure, defined as revisional bariatric/metabolic surgery, represents an effective therapeutic option, but it requires a re-operation, which often has increased morbidity in comparison to primary intervention [8]. The European Association for the Study of Obesity (EASO) and other medical associations recommend, after excluding surgical factors, reinforcement of nutritional, physical, and psychological counseling or pharmacotherapy in non-responder patients [9]. In fact, the nutritional approach for patients experiencing weight regain is still debated. In recent years, considerable attention has been paid to very low-calorie ketogenic diets (VLCKDs) as a possible strategy for obesity management [10, 11]. VLCKDs provide, in addition to a very-low-calorie content (about 500–800 kcal/day), a severe reduction in carbohydrates (< 50 gr/day), 1.2–1.5 g of protein/kg of ideal body weight, and 15–30 g of fat/day. The reduction in carbohydrate intake under the above reported conditions leads to ketone synthesis [12]. Ketone bodies are then utilized as fuel by several extrahepatic tissues, such as the central nervous system, skeletal muscle, and the heart, with potential benefits for several diseases [13–15]. Evidence also suggests significant amelioration of metabolic parameters and inflammation markers [16, 17]. Adherence to VLCKD seems to be facilitated by the effect of ketone bodies in controlling appetite and improving food control [18]. All these data might make VLCKDs a valuable option for the management of poor responder subjects after bariatric surgery. However, only limited data [19] are available on the safety, tolerability, and efficacy of this nutritional approach following bariatric surgery. Therefore, the aim of our study was to investigate the efficacy, feasibility, and tolerability of VLCKDs in a cohort of subjects with poor responses after bariatric surgery.

## Methods

This study was conducted at the Obesity and Dietetic Center, in Garibaldi Hospital, Catania, Italy among 26 non-diabetic obese adult patients, who had been treated with bariatric surgery (sleeve gastrectomy, SG, or mini-gastric bypass, MGBP) and experienced poor response.

Poor response after bariatric surgery was categorized as either IWL or WR. IWL was defined as an initial weight loss of less than 50% of excess weight loss (EWL), WR was defined as gain of at least 15% of the weight lost after bariatric surgery [3]. WR was calculated using the following formula: (current weight − nadir weight)/ (pre-bariatric weight − nadir weight) × 100 [20].

### Study protocol

This real-life observational prospective study was performed in the multidisciplinary clinical center, which includes expert physicians and a trained dietician. The center is specialized in the medical management of obesity, with specific expertise in VLCKD programs and the management of post-surgical bariatric patients.

All patients were Caucasian and the VLCKD program was offered for all 26 patients (18 females and 8 males). The exclusion criteria for the study were pregnancy or breastfeeding, acute illness or infections, and comorbidities limiting the efficacy and safety of the treatment (type 1 diabetes, renal or hepatic insufficiency, recent stroke or myocardial infarction, nephrolithiasis, abuse of drugs or alcohol, eating disorders, severe depression or any other psychiatric disease, neoplasia, arrhythmic heart diseases and heart failure, respiratory failure, and cortisone therapies). Additionally, secondary endocrine causes of obesity were excluded (hypo-thyroidism and hyper-cortisolism).

Among all the patients, 2 subjects declined to participate in the protocol, 1 patient had exclusion criteria (heart failure), and 1 patient withdrew before starting treatment. Altogether, 22 patients (15 females and 7 males) were eligible for the study; in detail, 12 patients had undergone to MGBP and 10 patients to SG. Pre- and post-surgical clinical characteristics of patients according to the bariatric procedure are represented in Supplementary Table 1.

The final study sample was composed of 10 patients (6 SG and 4 MGBP) who experienced IWL (EWL% 34.9 ± 10.1) and 12 patients (4 SG and 8 MGBP) with WR (46.1 ± 27.6%WR) after 6.5 ± 2.8 years from the first bariatric procedure.

The Ethical Committee Catania 2 approved the study (87/CECT2, 15/02/2022). All the patients gave written informed consent, and investigations were performed in accordance with the principles of the Declaration of Helsinki.

### Nutritional intervention

The nutritional program consisted of three dietary stages: (1) active stage (6 weeks), (2) re-education stage (5 weeks) and (3) maintenance stage (6–8 weeks) (Supplementary Table 2). Before starting the nutritional intervention, each patient underwent an electrocardiogram (ECG) to exclude any cardiac arrhythmia or alterations.

1. Active stage consisted of a VLCKD (570–670 kcal/day) with a carbohydrate intake lower than 10% (10–14 gr), a protein intake of 48% (69–78 gr), and a lipid intake of 42–47% (27–35 gr) as represented in Supplementary Table 2. For two weeks, the patients consumed only four substitutive meals vegetables with low glycemic indexes, and 10 g of olive oil per day. Specifically, to guarantee an adequate protein intake, the diet was composed of 4 daily meals for women (breakfast, lunch, dinner, mid-morning snack or mid-afternoon snack) and 5 daily meals for men (breakfast, mid-morning snack, lunch, mid-afternoon snack, and dinner). Later, natural protein (e.g., meat, eggs, or fish) was introduced to the lunch and dinner preparations instead of the protein preparation for breakfast and snacks. Ketosis was maintained during the active stage, which lasted 6 weeks. Supplementations of minerals (sodium, potassium, magnesium, and calcium), vitamins (complex B, C, and E), and omega-3 fatty acids were provided. Vitamins usually recommended after surgery not included in those provided were continued by the oral supplementation [21]. A water intake of at least 2 L/day was recommended.
2. In the second re-education stage comprising of VLCD 800–1000 kcal/day, the amount of carbohydrates progressively increased with the introduction of a portion of dairy products or fruits with a low glycemic index for breakfast and snacks.

In the first and second stages, intense aerobic activity was not recommended: the patients were invited to maintain an active lifestyle and instructed to have low-intensity physical activity [22].

(C) The last maintenance stage consisted of a progressive increase in the caloric content and nutrients with the introduction of legumes, cereals, bread, and pasta until they were able to maintain a balanced diet with a nutritional intake of about 1200 kcal. At least 150 min of moderate-intensity aerobic physical activity throughout the week was recommended in this phase which lasted 6–8 weeks.

The whole 3 stages program lasted 17–19 weeks. At the end of the protocol, all patients return to a hypocaloric Mediterranean diet. All patients voluntarily referred to Therascience Lignaform (Monaco, France) for the purchase of substitutive meals.

### Anthropometric measurements

All the patients underwent a physical examination at baseline and at the end of every phase. Height, body weight (BW), waist circumference (WC), hip circumference (HC) and arm circumference (AC) were assessed by a registered dietitian with clinical experience according to World Health Organization standards [23].

### Body composition

Body composition was assessed using bioelectrical impedance analysis (BIA) carried out on the participants after 3 h fasting using the TANITA MC 180 MA measuring station. Fat mass (FM), fat-free mass percentage (FFM), and total body water (TBW), extracellular water (ECW) and intracellular water (ICW) were evaluated.

### Muscular strength

Muscular strength was measured with a Jamar handgrip dynamometer (JAMAR®, Duluth, Minnesota). The grip size of the dynamometer was adjusted until the second joint of the index finger was at a 90-degree angle on the handle (90° flexion between the proximal and middle phalangeal joint). After providing instructions and demonstration, the patients, in the standing position, were invited to squeeze the hand grip as hard as possible for at least 3 s. The patients had to perform three efforts with the dominant limb, and the highest score was recorded as peak grip strength (kg).

### Blood analyses

Blood specimens were obtained after an overnight fast, and all laboratory analyses were performed in a single clinical laboratory according to standard procedures using commercially available kits. A comprehensive metabolic and lipid panel was carried out at baseline and at the end of the VLCKD (active stage). This included measurements of glucose, insulin, total cholesterol, high-density lipoprotein (HDL) cholesterol, triglycerides (TG), alanine transaminase (AST), aspartate transaminase (ALT), gamma-glutamyl transferase (GGT), albumin, creatinine, uric acid, sodium, and potassium. Low-density lipoprotein (LDL) cholesterol was calculated using the Friedwald formula (total cholesterol–HDL)–(TG/5). The glomerular filtration rate was calculated using the creatinine equation of the Chronic Kidney Disease Epidemiology Collaboration (CKD-EPI). To investigate potential nutritional deficiencies, serum iron, vitamin B12, folic acid, calcium, phosphorus, and 25-hydroxyvitamin D3 [25(OH)D] were measured.

### Ketosis

Ketosis was determined, after an overnight fast, by measuring the ketone bodies in capillary blood using a portable meter (GlucoMen LX Sensor®). All the patients were instructed to measure ketones once a week during the entire VLCKD program.

### Hunger, satiety, and control of eating

Self-reported appetite and fullness sensations during the three phases were assessed using 100 mm visual analog scales (VAS) [24, 25]. Selected appetite and craving-related items of the Control of Eating Questionnaire (COEQ) [26] were administered to measure hunger, satiety, food craving, and control of eating during all visits.

### Statistical analysis

The values were provided as the mean ± standard deviation. Differences in clinical characteristics and metabolic parameters were compared by paired parametric and non-parametric tests. The significance limit was set at *p* values of < 0.05. The data analyses were performed using the JMP statistical package software (version 10.0).

The sample size was calculated based on a previous study [19] which demonstrated a significant weight loss in all patients studied who experienced IWL or WR after bariatric surgery. Accordingly, it was established that at least 17 number of pairs were needed to achieve a power of 80% and a level of significance of 5% (two sided) for detecting a mean of the differences of 5–10% weight loss between pairs, assuming the standard deviation of the differences to be 10.

## Results

All the recruited patients had a mean age of 50.3 ± 10.3 years, a BW of 100.4 ± 17.7 kg, and a BMI of 37.5 ± 5.5 kg/m^2^. The anthropometric measurements, body composition, and metabolic parameters are reported in Tables 1, 2.

**Table 1:** Anthropometric measurements and body composition parameters before and after each dietary phase

	Basal	Active stage	Re-education stage	Maintenance stage
BW (kg)	100.4 ± 17.7	91.5 ± 16.9^*^	87.2 ± 15.3^*^^	85.9 ± 15.2^*§^
BMI (kg/m^2^)	37.5 ± 5.5	34.3 ± 5.4^*^	32.6 ± 4.7^*^^	32.1 ± 4.6^*§^
WC (cm)	117.5 ± 13.9	104.7 ± 18.2^*^	105.6 ± 14.0^*^^	102.7 ± 12.5^*§^
HC (cm)	121.6 ± 13.1	116.6 ± 13.2^*^	112.1 ± 10.9^*^^	110.4 ± 9.8^*§^
AC (cm)	38.7 ± 4.9	37.1 ± 4.1^*^	35.6 ± 4.2^*^^	36.3 ± 3.9^*^
MS (Kg)	26.0 ± 6.7	25.4 ± 6.8	25.3 ± 6.1	25.2 ± 6.3
FM (kg)	40.7 ± 10.8	35.0 ± 10.6^*^	31.0 ± 8.5^*^^	30.1 ± 8.7^*^^
FFM (kg)	59.7 ± 10.4	56.5 ± 9.8^*^	56.2 ± 9.8^*^	55.7 ± 7.7^*^
TBW (kg)	42.5 ± 7.3	39.7 ± 6.2^*^	39.0 ± 7.8^*^	39.7 ± 7.3^*^
ICW (kg)	24.6 ± 4.2	23.0 ± 3.6^*^	22.5 ± 4.5^*^	22.9 ± 4.2^*^
ECW (kg)	17.9 ± 3.1	16.8 ± 2.6^*^	16.5 ± 3.3^*^	16.8 ± 3.1^*^

Data are shown as means ± standard deviation (SD). *p < 0.05 vs. basal, ^*p* < 0.01 vs. active, §*p* < 0.05 vs. re-education

*BW* body weight, *BMI* body mass index, *WC* waist circumference, *HC* hip circumference, *AC* arm circumference, *MS* muscular strength, *FM* fat mass, *FFM* fat-free mass

**Table 2:** Blood biochemical parameters at baseline and at the end of active stage

	Basal	End of active stage
Glucose (mg/dL)	88.3 ± 11.2	79.6 ± 7.6*
Insulin (μU/mL)	11.0 ± 9.3	6.5 ± 3.9*
Total cholesterol (mg/dl)	202.5 ± 42.2	177.5 ± 37.5*
HDL Cholesterol (mg/dl)	53.2 ± 10.8	47.6 ± 10.4*
Triglycerides (mg/dl)	109.4 ± 49.8	82.9 ± 31.3*
LDL Cholesterol (mg/dl)	127.4 ± 34.0	113.7 ± 28.8*
AST (mg/dL)	25.2 ± 6.8	26.4 ± 8.5
ALT (mg/dL)	27.2 ± 10.2	27.3 ± 11.7
Gamma GT(UI/L)	22.7 ± 8.2	19.7 ± 12.1*
Creatinine (mg/dl)	0.70 ± 0.1	0.72 ± 0.1
eGFR CKD (ml/min/1.73 m^2^)	104.6 ± 11.3	104.5 ± 15.2
Sodium (mmol/L)	138.7 ± 1.9	138.4 ± 1.9
Potassium (mmol/L)	4.2 ± 0.2	4.2 ± 0.3
Uric Acid (mg/dL)	5.3 ± 1.2	5.8 ± 1.5
Calcium (mg/dL)	9.1 ± 1.8	9.7 ± 0.5
Phosphorus (mg/dL)	3.5 ± 0.5	3.6 ± 0.4
Albumin (g/dL)	4.3 ± 0.3	4.4 ± 0.2
Iron(mcg/dl)	73.8 ± 23.2	66.9 ± 23.4
Vitamin B12 (pg/ml)	366.1 ± 120.2	426.1 ± 168.9
Folic acid (ng/ml)	7.3 ± 4.1	9.3 ± 4.5
25(OH)D (ng/ml)	27.9 ± 19.2	32.9 ± 17.7

*p < 0.05 vs. basal. Data are shown as means ± standard deviation (SD). *LDL* low-density lipoprotein, *HDL* high-density lipoprotein, *eGFR* estimated glomerular filtration rate, *CKD* Chronic kidney disease

The capillary blood β-hydroxybutyrate concentration increased during the active stage (1.0 ± 0.6 mmol/L).

A significant (*p* < 0.05) weight loss was observed, in respect to the baseline values, at the end of the active stage (8.7 ± 3.3%), the re-education stage (12.8 ± 4.4%) and the maintenance stage (14.1 ± 4.8%). No difference in weight loss during the nutritional program between SG and MGBP patients was observed either in patients who experienced WR or IWL.

**Fig. 1:**
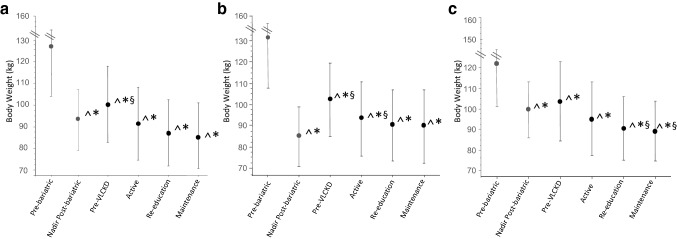
Body weight in the entire cohort (**a**), in patients with WR (**b**) and in patients with IWL (**c)** **^***p* < 0.005 vs pre-bariatric; §*p* < 0.005 vs nadir post-bariatric; *p < 0.005 vs pre VLCKD

Surgical weight history as follows preoperative weight, nadir weight, and weight at regain is presented in Fig. 1a. During the VLCKD program, patients who had experienced WR lost nearly the same amount of body weight as that previously regained after bariatric surgery, and at the end of the nutritional program, their body weight was not significantly different from that at nadir (88.1 ± 14 vs 92.8 ± 17, *p* = 0.09) while patients who had experienced IWL reached a body weight significantly lower than that obtained at post-bariatric surgery weight nadir (89.3 ± 14.4 vs 99.6 ± 14, *p* = 0.0004) (Fig. 1b, c).

Additionally, BMI, WC, and HC were significantly reduced at the end of all three stages (Table 1). Most of the initial body weight lost was FM with a minor reduction in FFM mostly due to total body water loss, both intra and extracellular. The measured muscular strength was not significantly different from the baseline at any time during the study.

### Biochemical outcomes

At the end of the active stage, patients obtained a relevant amelioration of their metabolic and lipid profiles with a significant reduction in fasting glucose and insulin, total cholesterol, HDL, triglycerides, and LDL cholesterol (Table 2).

A significant decrease in GGT levels was observed, whereas creatinine, eGFR, AST, ALT, uricemia, albumin, and electrolytes were unchanged (Table 2). Finally, the levels of serum iron, vitamin B12, folate, calcium-phosphate homeostasis, and 25 (OH) D remained stable. No significant side effects or gastrointestinal disturbance involving nausea/vomiting or diarrhea were reported.

### Hunger, eating, and food craving

**Fig. 2:**
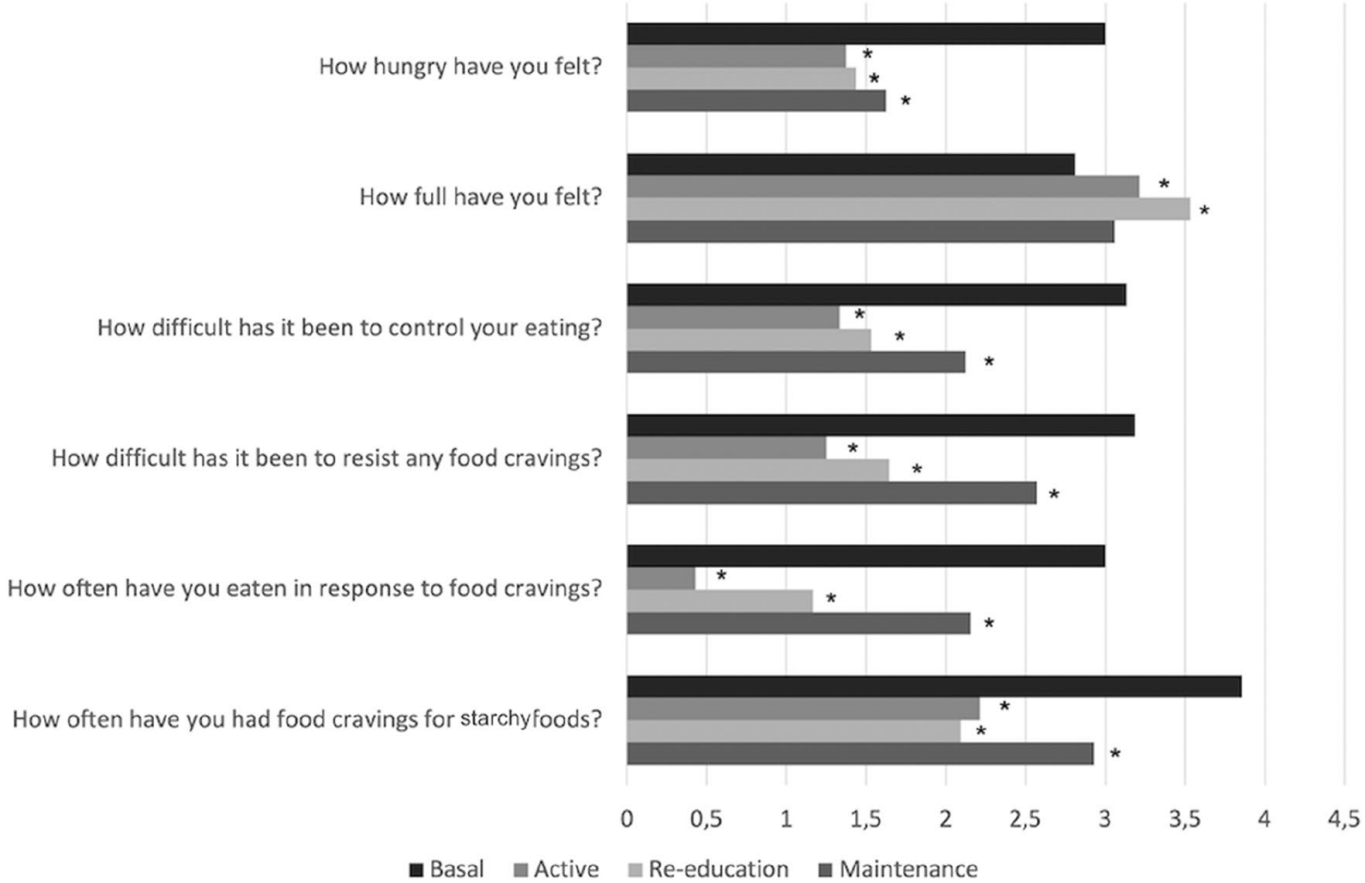
Change in hunger, eating, and food craving-related items from the Control of Eating Questionnaire during the protocol. *p < 0.05 vs basal

Subjective hunger, fullness, control of eating, and food craving were evaluated by VAS scores [19]. Patients reported reduced hunger and increased ability to control eating and tolerate food craving, reducing eating as a response to it. Additionally, patients especially experienced reduced food cravings for starchy foods. During the re-education stage, patients described increased feelings of fullness (Fig. 2).

## Discussion

In this prospective study on 22 non-diabetic adult patients with obesity, we observed for the first time the dietary approach of VLCK is beneficial for post-bariatric surgical treatment among those with poor response. Significant weight loss reaching 14%, mostly due to FM, while preserving muscular strength was detected. Additionally, improvements in the metabolic profiles were observed. The significantly beneficial changes in nutritional behaviors were observed, as well as nutritional regimen was well tolerated.

To our knowledge, this is the first prospective study assessing the efficacy, feasibility, and tolerability of VLCKDs in the management of patients with IWL or WR. However, the management of poor response after bariatric surgery remains an issue of debate. This dietary approach has been recommended for preoperative weight loss in patients who are candidates for bariatric surgery and require a reduction in liver volume and visceral adiposity [17]. However, only one retrospective study evaluated its use after bariatric surgery [19]. In our study, we included 22 subjects with two different bariatric procedures (sleeve gastrectomy and mini-gastric bypass) who experienced poor responses nearly 6 years after the surgery. All patients obtained more than 5% of weight loss after the VLCKD phase and these results were also confirmed after the maintenance phase; at the end of it, 86% of patients had lost more than 10% of their body weight. Weight loss was mostly due to a decrease in FM, as well as a reduction in visceral adiposity. The expected FFM loss was mainly due to total body water loss, probably because of the intense diuresis, and was accompanied by the preservation of muscular strength. These results are in line with those reported for the effect of VLCKD on muscular mass [27]. It has been demonstrated that adequate protein intake during the first weeks of a ketogenic diet supplies the amino acids for gluconeogenesis and prevents muscle loss. This effect is critical in post-bariatric patients who already experienced the loss of FFM after surgery. The loss of muscle mass may represent one of the possible contributors to WR, causing a decrease in resting energy expenditure that may limit weight loss over time [28]. The VLCKD allowed WR patients to lose the amount of body weight regained after bariatric surgery and IWL patients to obtain greater weight loss than that at nadir post-surgery. It also showed additional benefits in terms of improvement of glycemic, insulinemic and lipid profiles. A significant reduction in glycemic levels could already be observed during the first days of the VLCKD. Although an improvement in triglyceride levels is clearly attributed to the VLCKD, an increase in LDL cholesterol has been described initially in some studies [29]. In our population, as well as in other reported populations, a significant reduction in LDL cholesterol levels, as well as triglycerides, was observed. The differences reported in some studies could be related to variations in the quality and fat content of the diet composition. An isocaloric ketogenic diet, generally used for epilepsy or other diseases not requiring weight loss, is high in fat to maintain a normal caloric intake and, therefore, can have a negative impact on lipid profiles [30].

Regarding the subjective feelings of appetite and control of eating, ketone bodies seem to have an appetite-suppressing effect [18, 31]. The COEQ results showed a significant reduction in hunger and food craving and a significant improvement in control of eating during the nutritional program. Patients also reported a significantly greater fullness when they started to reintroduce all the nutrients in the re-education; this could be due to their reduced gastric capacity resulting from surgery but also to the decreased levels of the orexigenic hormones such as ghrelin and enhanced secretion of satiety gut hormones such as glucagon-like peptide 1 (GLP-1) following bariatric surgery. Several studies have demonstrated the safety of the VLCKD in patients with obesity and with mild kidney failure [32]. Our data demonstrated the safety of the VLCKD in bariatric patients regarding renal and liver function with no change in the estimated glomerular filtration rate (eGFR), uricemia, electrolytes, and transaminases. As is known, bariatric patients, especially those who undergo a malabsorptive procedure, can develop nutrient deficiencies and need to adhere to multivitamins and mineral supplementation. Additionally, during the VLCKD, mineral supplementation is recommended to avoid nutrient deficiencies caused either by a very restrictive caloric intake or the exclusion of many kinds of vegetables and fruits [17]. In our study, patients continued the supplementation of multivitamins usually used after surgery in the VLCKD. This strategy allowed the patients to maintain normal levels of serum iron, vitamin B12, folate, and 25(OH)D. A good tolerability was reported without the occurrence of gastrointestinal side effects including nausea, vomiting, or diarrhea. This represents a critical issue in bariatric surgery, where patients frequently experience gastrointestinal disturbances related to anatomical and physiological modifications following the surgery [33].

This study has some limitations: the short follow-up period does not allow us to give univocal conclusions on the long-term impact of this dietary approach and the small sample size. Further larger studies would be required to confirm our results.

As a complex and chronic disease, obesity needs a lifelong treatment with sequential use of different strategies. Additionally, the treatment options for patients with IWL or WR following bariatric surgery are limited. Revisions of bariatric procedures, when applicable, have higher rates of complications than the primary intervention. Weight loss medications may provide a safer alternative for weight management in these patients [34], but these are still underutilized, although they are recommended by the European Association for the Study of Obesity (EASO) after reinforcement of nutritional, physical, and psychological advice. As a nutritional approach, VLCKDs might represent a useful tool for patients with IWL or WR after bariatric surgery as a part of a multimodal and sequential approach, taking into account also pharmacotherapy, cognitive behavior therapy, and, if necessary, revisional surgery, in the treatment of a complex chronic disease such as obesity.

## Conclusions

This study demonstrated the efficacy, feasibility, and tolerability of VLCKDs in patients with poor response after bariatric surgery. These data on VLKDs are promising as a possible strategy for weight management in post-bariatric surgery.

## Supplementary Information

Below is the link to the electronic supplementary material.

Supplementary file1 (DOCX 25 KB)

## Data Availability

The dataset analyzed during the current study is available from the corresponding author on reasonable request.
