# Supplementary material for: Efficacy, feasibility and tolerability of ketogenic diet for the treatment of poor response to bariatric surgery
Source: J Endocrinol Invest. 2023 Feb 21;46(9):1807–14. doi: 10.1007/s40618-023-02034-2 (PMC10371952; doi:10.1007/s40618-023-02034-2)
Supplement: Supplementary file 1 — Supplementary file1 (DOCX 25 KB) [file 40618_2023_2034_MOESM1_ESM.docx]

SUPPLEMENTARY TABLES

|  | **MGBP**  n=12 (7 F/5 M) | | **SG**  n= 10 (8 F/2M) | |
| --- | --- | --- | --- | --- |
|  | **Pre-bariatric** | **Nadir**  **Post-bariatric** | **Pre-bariatric** | **Nadir**  **Post-bariatric** |
| BW (kg) | 137.4±24.9 | 98.5±15.9* | 116.4±11.4 | 87.4±12.4* |
| BMI (kg/m^2^) | 48.2±4.2 | 35.7±5.9* | 43.9±4.8 | 33.9±6.7* |
| Hypertension (%) | 91.6% (n=11) | 8.3% (n=1) | 80% (n=8) | 10% (n=1) |
| Dyslipidemia (%) | 50% (n=6) | 8.3% (n=1) | 30% (n=3) | 20% (n=2) |
| IFG and/or IGT | 58.3% (n=7) | 0% | 10% (n=1) | 0% |

**Supplementary Table 1.** Clinical characteristics of patients according to bariatric procedure (mini gastric bypass, MGBP and sleeve gastrectomy, SG) before and after surgery.

F: females, M: males; BW: body weight, BMI: body mass index, IFG: impaired fasting glucose; IGT: impaired glucose tolerance.

Data are shown as means ± standard deviation (SD).

*p<0.0001 vs pre-bariatric post-bariatric.

|  | ACTIVE STAGE | | RE-EDUCATION STAGE | | MAINTENANCE STAGE | |
| --- | --- | --- | --- | --- | --- | --- |
|  | F | M | F | M | F | M |
| Intake kcal/day | 570-700 | 670-800 | 800-900 | 900-1000 | 1000-1100 | 1100-1200 |
| Carbohydrates | <10%,  10-15 gr | < 10%,  10-18 gr | < 15-17%  32-40 gr | < 15-17%  40-45 gr | 36-45%  96-132 gr | 36-45%  105-144 gr |
| Protein | 48-43%  69-75 gr | 47-45%  78-90 gr | 38-35%  75-78 gr | 40-35 %  90-87 gr | 28-25%  70-69 gr | 28-25%  77-75 gr |
| Lipid | 42-47%  27- 40 gr | 47-48%  34-43 gr | 45-48%  41-48 gr | 45-48%  45-53 gr | 36-30%  40-37 gr | 36-30%  44-40 gr |

**Supplementary Table 2.** Caloric intake and macronutrient percentage in the different phases of Nutritional Intervention
